# Human Leukocyte Antigen (HLA) at the Root of Persistent Antigens and Long COVID

**DOI:** 10.29245/2578-3009/2025/1.1257

**Published:** 2025-01-06

**Authors:** Apostolos P. Georgopoulos, Lisa M. James, Phillip K. Peterson

**Affiliations:** 1 Brain Sciences Center, Minneapolis Veterans Affairs Health Care System, Minneapolis, MN, USA; 2 Department of Neuroscience, University of Minnesota Medical School, Minneapolis, MN, USA; 3 Department of Psychiatry, University of Minnesota Medical School, Minneapolis, MN, USA; 4 Department of Medicine, University of Minnesota Medical School, Minneapolis, MN, USA

**Keywords:** Antigen persistence, Human Leukocyte Antigen, Long COVID-19

## Abstract

Here we offer a perspective on recent findings of persistent SARS-CoV-2 antigens in Long COVID^1^ through the lens of immunogenetic risk and protection, namely in the context of the fundamental role of Human Leukocyte Antigen (HLA) in eliminating viral infections. Specifically, we attribute the persistence of viral antigens to the lack or weak protection conferred by HLA against SARS-CoV-2 in individuals carrying HLA alleles with low binding affinities to the virus. We suggest that determining the HLA Class I and II makeup of Long COVID patients will provide valuable new information in elucidating the cause for antigen persistence underlying the development of Long COVID and pave the way for successful interventions.

A recent study^1^ documented evidence of SARS-CoV-2 antigens circulating in the blood up to 14 months post COVID-19 infection and reported an association between SARS-CoV-2 antigen-positivity and post-acute sequelae (PASC) of COVID-19 involving several symptom domains. We attribute this antigen persistence to the immunogenetic makeup of the patients, according to the following considerations. With respect to SARS-CoV-2 and other viral insults, the immediate defense lies with the Human Leukocyte Antigen (HLA) Class I (HLA-I) system which typically eliminates the virus during the first ~10 days of infection^2^, after which the viral infection, if it persists, tends to become chronic. An important property of the HLA-I system is that its molecules are expressed in all nucleated cells (with some exemptions, e.g. neurons) and are therefore available almost everywhere in the body, providing a wide coverage of potential points of insult. The HLA-I molecules code for cell-surface proteins that bind with 8–10 amino acid (AA) long peptides (epitopes) of the viral protein(s) to form a peptide-HLA-I complex (pHLA-I) which is presented to CD8+ cytotoxic T cells to signal the destruction of the infected cell^3^. Now, this will work if, and only if, a pHLA-I complex is successfully formed. Unfortunately, this is not guaranteed for everyone because an individual carries only 6 HLA-I alleles (2 of each 3 classical HLA-I genes: A, B, C), which means that the chance of successful formation of a pHLA-I complex, and hence the chance for elimination of the infected cell, is not necessarily guaranteed.

Fortunately, the HLA system has evolved over millions of years to promote survival from viral and other insults, endowing human populations with HLA molecules covering a large variety of such insults. However, although this is true for the population as a whole, the successful protection of a particular individual still hinges on the specific HLA makeup of that individual. Moreover, it should be noted that the formation of the pHLA-I complex is, biophysically, a probabilistic process, the successful outcome of which is determined by the strength of binding affinity of the antigen peptide-epitope to a particular HLA-I molecule: the higher the binding affinity, the higher the chance that a stable pHLA-I complex will be formed. Thus, the binding of an antigen epitope to an HLA-I molecule is the first and necessary step in eliminating an infected cell, an outcome that critically depends on the pHLA-I binding affinity.

The second line of HLA defense is mediated by the HLA Class II (HLA-II) system which enables the production of antibodies against foreign antigens^3^. Each individual carries 6 HLA-II alleles (2 of each 3 classical genes: DPB1, DQB1, DRB1). The initial steps are very similar to the HLA-I case described above, namely (a) the successful formation of a pHLA-II complex (where peptides-epitopes are typically 15 AA long), (b) presentation of the pHLA-II complex to CD4+ T cells, and (c) production of antibodies against the antigen by B cells. A major difference between HLA-I and HLA-II systems is that HLA-II molecules are expressed only in professional antigen presenting cells (APC), including dendritic cells, macrophages, and B cells. Production of antibodies takes ~2 weeks. Antibodies can neutralize the harmful effect of a virus following an infection^4^, thus aiding further reduction of viral damage, and can also protect against viral attacks in the future, the mechanism by which vaccines work. Thus, the strength of binding affinity of viral protein peptides to HLA-I/II for eliminating infected cells (HLA-I) and production of antiviral antibodies (HLA-II) is the first, essential and necessary step against a viral infection in otherwise immunocompetent individuals. By applying these considerations to SARS-CoV-2 infection (and the disease caused by it COVID-19), we have the following two main scenarios, outlined in Fig. 1, as examples of non-fatal disease outcomes.

In summary, we attribute the persistence of SARS-CoV-2 antigens in Long COVID to inadequate HLA-I/II protection. Several studies have documented associations of HLA and COVID-19^5^, as we predicted early on^6^, but, to our knowledge, there is no such study focused on Long COVID. The study of Swank et al.^1^ offers a golden opportunity to uncover the main reason for SARS-CoV-2 antigen persistence in Long COVID by determining the HLA-I/II genotype of their study participants and exploring the association of this genotype to specific antigen persistence. A further step would be to estimate the binding affinity between the SARS-CoV-2 spike, S1 and N antigens identified in PASC patients^1^ and the HLA-I/II molecules carried by the patients, an estimation that can be obtained *in silico*^7^. We predict that the HLA molecules of the PASC patients will have low binding affinities to SARS-CoV-2 spike glycoprotein, allowing the persistence of its fragments and thus rendering the patients more vulnerable to developing PASC and Long COVID. In a way, this resembles the positive association found between the observed clinical effectiveness of vaccines against different SARS-CoV-2 variants of concern and the estimated binding affinities of their spike glycoproteins to HLA-II alleles^8^, where binding affinities predicted accurately the observed vaccine clinical effectiveness (Fig. 2 in ref. 8).

It should be noted that the present case of persistent SARS-CoV-2 antigens is actually a subset of a larger family of persistent harmful antigens involved in chronic disease, including the peptidoglycan in the case of chronic Lyme arthritis^9^, the anthrax vaccine antigen in Chronic Multisymptom Illness (formerly Gulf War Illness)^10^, and potentially other persistent antigens in Myalgic Encephalomyelitis / Chronic Fatigue Syndrome (ME/CFS)^11^. It is important to notice that such persistent antigens have also been shown to be harmful in vitro^12–14^ and in vivo^15,16^. Antigen persistence has had a long history^17,18^ but it is only recently that harmful antigen persistence has begun to be recognized as underlying a variety of chronic, multisymptom conditions^11^. More importantly, the recognition of the critical role of HLA immunogenetic makeup in allowing antigen persistence^19^ will help assess the risk that an individual might develop Long COVID, based on the strength of binding affinity of SARS-CoV-2 epitopes to the individual’s 12 HLA-I/II molecules to form stable pHLA-I/II complexes. This would also account for the variation in severity of Long COVID symptoms. The same considerations with respect to HLA-II system apply to predicting the effectiveness of COVID vaccination^8^ in a specific individual and could account for the wide variation observed in vaccine effectiveness, as seen in the wide confidence intervals of clinical trials (Table 4 in ref. 8). In a way, the recognition of the critical role of the immunogenetic (HLA) makeup of an individual in allowing for, or protecting against, persistent antigens and Long COVID in that individual, is a good example of personalized medicine.

## Figures and Tables

**Figure 1.**
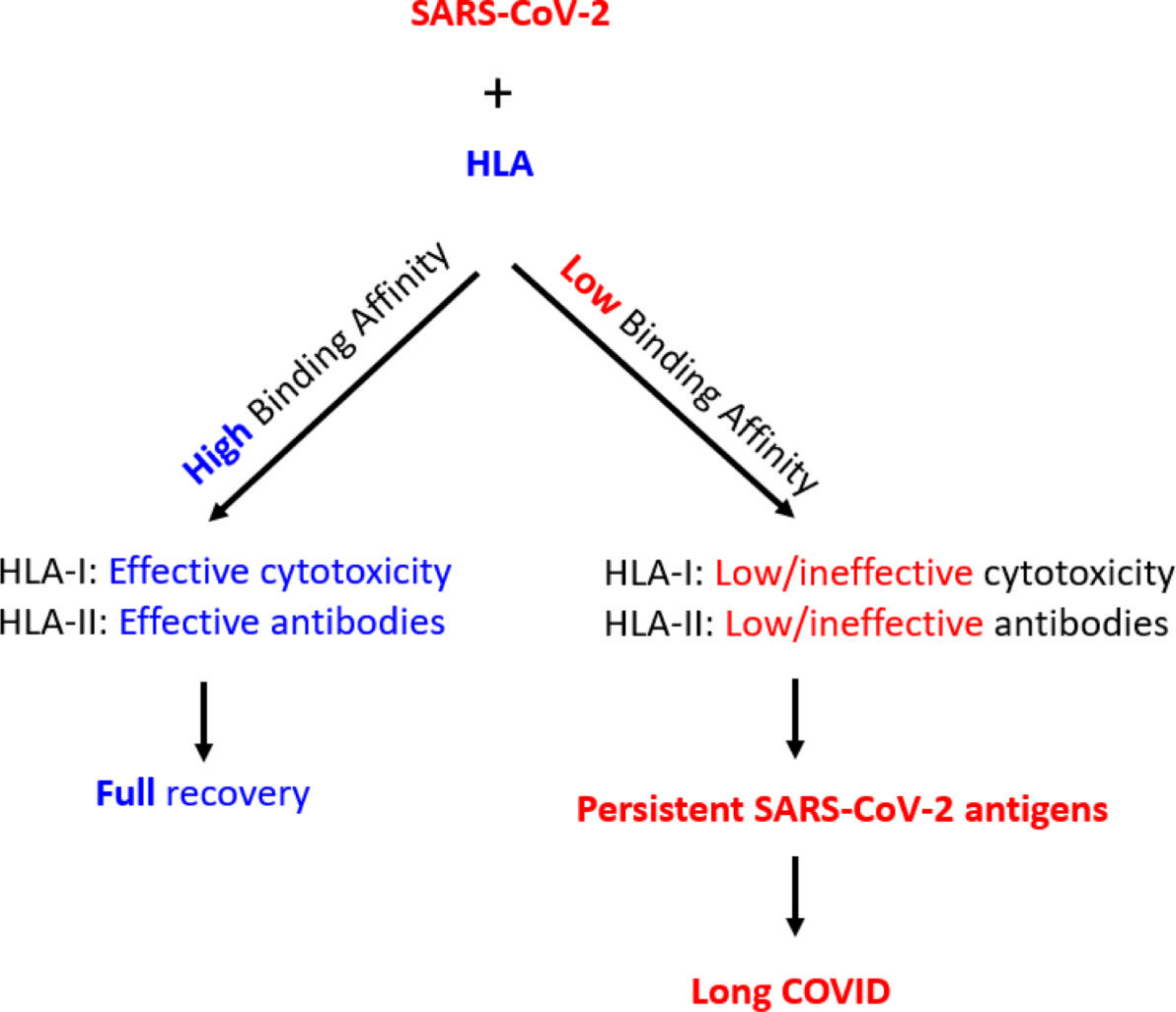
Schematic diagram to illustrate two possible outcomes of SARS-CoV-2 infection leading to full recovery (left panel; blue) or Long COVID (right panel; red). See text for details.
